# Staufen1 unwinds the secondary structure and facilitates the translation of fatty acid binding protein 4 mRNA during adipogenesis

**DOI:** 10.1080/21623945.2021.1948165

**Published:** 2021-07-05

**Authors:** Xiaodi Liang, Yi Jiao, Xueli Gong, Hao Gu, Nuerbiye Nuermaimaiti, Xuanyu Meng, Dihui Liu, Yaqun Guan

**Affiliations:** a State Key Laboratory of Pathogenesis, Prevention and Treatment of High Incidence Diseases in Central Asia, Department of Biochemistry and Molecular Biology, Preclinical Medicine College, Xinjiang Medical University, Urumqi, Xinjiang, China; b Department of Pathophysiology, Preclinical Medicine College, Xinjiang Medical University, Urumqi, Xinjiang, China; c Department of Laparoscopic Surgery, First Affiliated Hospital, Xinjiang Medical University, Urumqi, Xinjiang, China

**Keywords:** Staufen1, adipogenesis, fatty acid binding protein 4, lipid metabolism, RNA processing

## Abstract

Adipogenesis is regulated by genetic interactions, in which post-transcriptional regulation plays an important role. Staufen double-stranded RNA binding protein 1 (Staufen1 or *STAU1*) plays diverse roles in RNA processing and adipogenesis. Previously, we found that the downregulation of *STAU1* affects the expression of fatty acid-binding protein 4 (FABP4) at the protein level but not at the mRNA level. This study aimed to determine the mechanism underlying the regulation of FABP4 expression by STAU1, explaining the inconsistency between FABP4 mRNA and protein levels. We used RNA interference, photoactivatable ribonucleoside enhanced cross-linking and immunoprecipitation, and an adeno-associated virus to examine the functions of STAU1 in adipogenesis. Our results indicate that STAU1 binds to the coding sequences of *FABP4*, thereby regulating the translation of *FABP4* mRNA by unwinding the double-stranded structure. Furthermore, STAU1 mediates adipogenesis by regulating the secretion of free fatty acids. However, STAU1 knockdown decreases the fat weight/body weight ratio but does not affect the plasma triglyceride levels. These findings describe the mechanisms involved in STAU1-mediated regulation of *FABP4* expression at the translational level during adipogenesis.

## Introduction

The adipose tissue is a metabolic organ that plays critical roles in the regulation of energy homoeostasis and lipid metabolism [1,2]. Adipocyte differentiation involves different levels of gene expression [3,4]. During translation, the secondary structure of mRNA affects translation efficiency, and higher levels of RNA binding proteins regulate this process [5,6].

Staufen double-stranded RNA binding protein 1 (Staufen1/*STAU1*) is a double-stranded RNA (dsRNA)-binding protein that can recognize the Staufen binding sites (SBS) and affect the structure, translation efficiency, and degradation of mRNA [7–9]. Nearly all double-stranded RNAs are recognized and bound by STAU1 [10], the expression of which is upregulated during adipogenesis [11]. In our previous study, we demonstrated that nearly 3,000 different mRNA species are upregulated upon downregulation of STAU1 expression during adipogenesis. Recent studies have indicated that STAU1 can sense the secondary structure of mRNA and regulate translation [10].

Previously, we have shown that mRNAs of fatty acid-binding protein 4 (*FABP4*) possess SBS in their open reading frames; this finding indicates that STAU1 may regulate the expression of *FABP4* [12]. FABP4, also called adipocyte protein 2, is a lipid chaperone [13]. *FABP4* encodes the 14 kD protein FABP4, which binds to fatty acids and facilitates the transportation of fatty acids to different organelles [14,15]. During adipogenesis, *FABP4* is transcribed by different transcription factors such as peroxisome proliferator-activated receptor γ (*PPARγ*) and CCAAT/enhancer-binding protein α (*C/EBPα*) [16]. PPARγ can recognize a specific motif in the promoter of *FABP4* and enhance *FABP4* transcription. However, high levels of FABP4 can trigger the degradation of PPARγ, thereby downregulating PPARγ levels [17]; this indicates a post-transcriptional regulation mechanism that regulates the expression of *FABP4*.

In this study, we used siRNAs to downregulate the expression of STAU1 to evaluate its function in adipogenesis. Furthermore, we sought to elucidate the mechanisms underlying STAU1-mediated expression of FABP4.

## Results

### Inhibition of STAU1 downregulates the expression of FABP4

**Figure 1.**
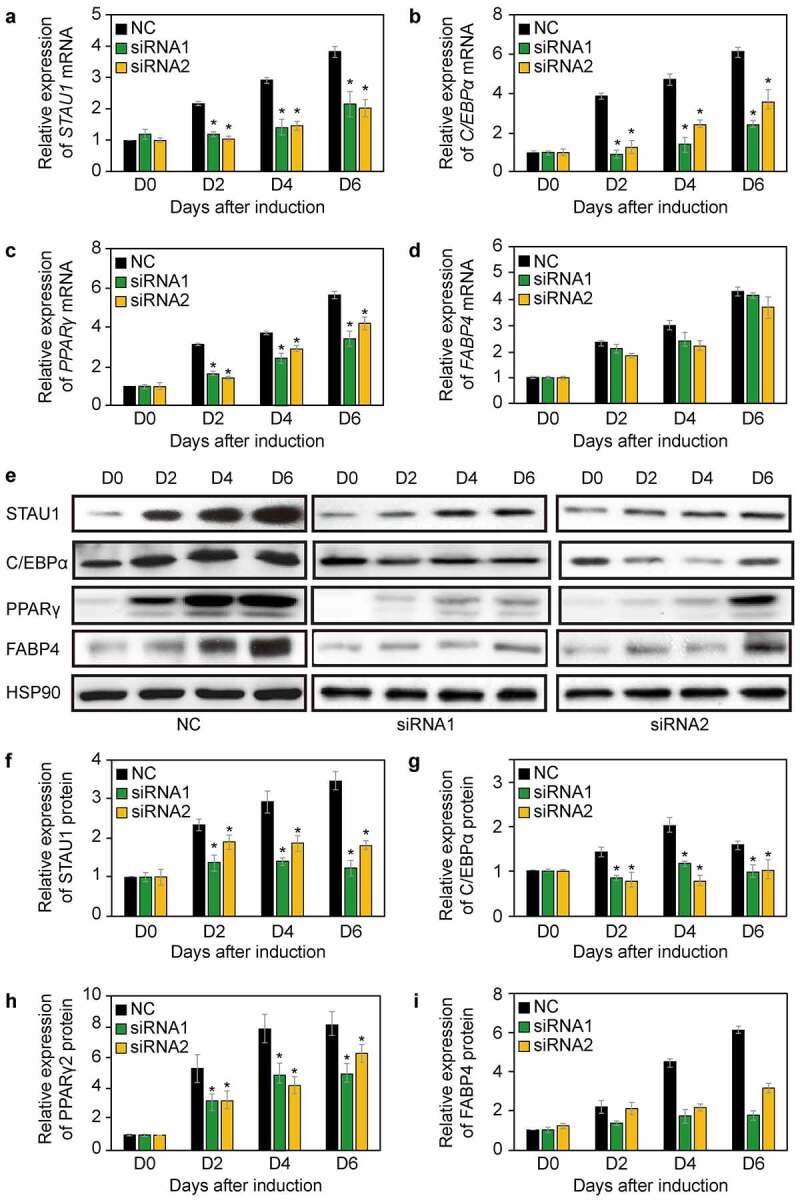
Expression of *STAU1, C/EBPα, PPARγ*, and *FABP4* after downregulation of *STAU1*. A-D: qPCR quantification of *STAU1, C/EBPα, PPARγ*, and *FABP4* mRNA levels on days 2, 4, and 6, as compared to the levels at day 0. E: Western blot was used to assess the levels of STAU1, C/EBPα, PPARγ, and FABP4 on days 2, 4, and 6; these levels were compared with those of negative control (NC). F-I: Quantification of STAU1, C/EBPα, PPARγ, and FABP4 expression levels assessed via western blotting on days 2, 4, and 6 and compared to the levels at day 0. The results are presented as the mean ± standard deviation (*P < 0.05, compared with NC)

3T3-L1 cells were induced with an adipogenic cocktail and then used to examine the differentiation of preadipocytes into adipocytes. siRNA1 and siRNA2 were used to downregulate *STAU1* as described previously [18]. Our results indicate that expression of *STAU1* is significantly downregulated by 1.7 ± 0.57-fold for siRNA1 and by 2.1 ± 0.48-fold for siRNA2 (*P* < 0.05, n = 3) compared with that of the negative control (NC) (Figure 1(a,e,f)). We next analysed the mRNA and protein levels of *C/EBPα*, PPARγ, and FABP4. Our results revealed that *C/EBPα* and *PPARγ* mRNA (Figure 1(b,c)) and protein levels (Figure 1(e,g,h)) are downregulated after the knockdown of *STAU1*. Moreover, the levels of FABP4 decrease, while its mRNA levels remain unchanged (Figure 1(d,e,i)). These results suggest that STAU1 may regulate the expression of *FABP4* at the post-transcriptional level.

### STAU1 binds to the SBS of FABP4 mRNA

**Figure 2.**
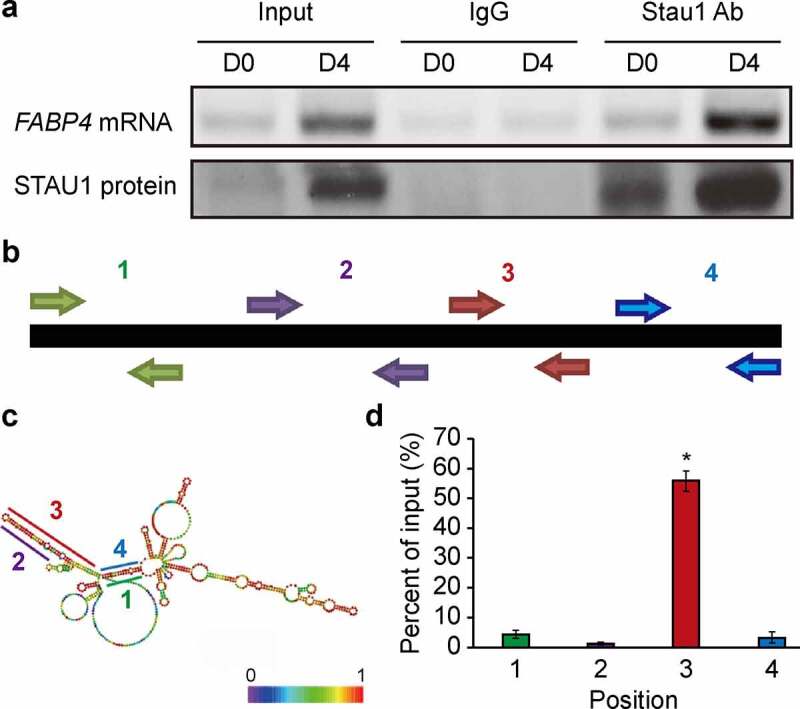
Binding of STAU1 to the CDS of *FABP4*. A: Results obtained using RT-PCR show the expression of *FABP4* mRNA precipitated by IgG or STAU1 antibody at 0- and 4-days post-induction. Western blot showing specific IPs of STAU1 at 0- and 4-days post-induction. B: The schematic shows PAR-CLIP primers designed for the different positions. C: Secondary structure of *FABP4* mRNA. Different putative SBS are indicated by different numbers. D: Results obtained via qPCR show the percentage of STAU1 binding to *FABP4* mRNA at different positions as compared with input *FABP4* mRNA. The results are presented as the mean ± standard deviation

We next used RIP to evaluate the binding of STAU1 to *FABP4* mRNA. After precipitating *FABP4* mRNA with a STAU1 antibody and IgG, we found that STAU1 could successfully pull down the *FABP4* mRNA, but not IgG (Figure 2(a)). A previous study has shown that STAU1 recognizes and binds to different SBS at different positions [10]. A typical SBS contains a stem-loop structure; therefore, we used an online database to predict the secondary structure of *FABP4* mRNA [19]. The nucleotide sequence of mouse *FABP4* mRNA, obtained from NCBI (NM_024406.3), contains 696 bases and a coding region of 106–504 base pairs. Analysis of the secondary structure of *FABP4* mRNA revealed at least four putative SBS, as shown in Figure 2(c). Among these four positions, 2–4 were located in the coding sequences (CDS) of *FABP4* mRNA; while, position 1 was located in the 5′ UTR. To further investigate which position is recognized by STAU1, we used photoactivatable ribonucleoside enhanced cross-linking and immunoprecipitation (PAR-CLIP) to proliferate different positions of *FABP4* mRNA via different primers (Figure 2(b)). Our results, obtained using qRT-PCR, show that STAU1 is able to recognize and bind position 3 (110–220) (Figure 2(d)). These findings suggested that STAU1 positively regulated the levels of FABP4 predominantly at the post-transcriptional level by directly binding to *FABP4* mRNA in the coding region.

### STAU1 facilitates the translation of FABP4

**Figure 3.**
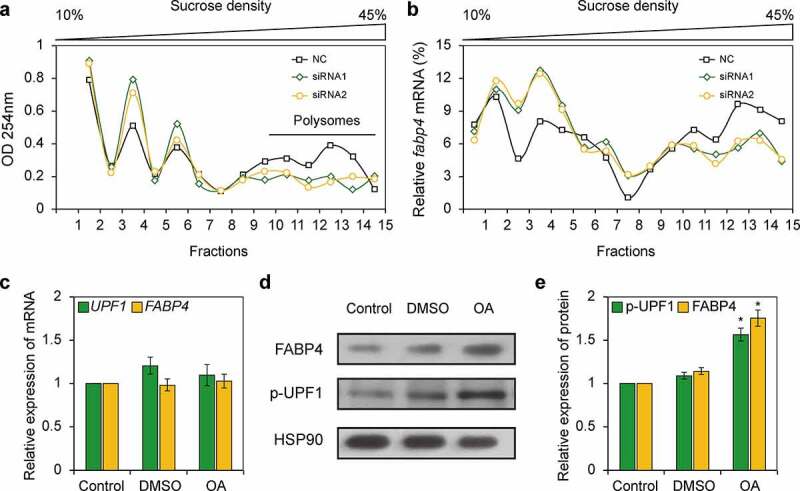
STAU1 upregulates the expression of FABP4 protein. A. Polysome profiling of 3T3-L1 cells on day 4 post-induction, obtained by the continuous reading of absorbance at 254 nm after downregulation of STAU1. B. Levels of *FABP4* mRNA were measured by qRT-PCR using 15 sucrose gradient fractions obtained after downregulation of STAU1 and on day 4 post-induction. C. 3T3-L1 cells were treated with 0.1 μM okadaic acid on day 4 post-induction; then, the *UPF1* and *FABP4* mRNA expressions were quantified by RT-qPCR. DMSO-treated 3T3-L1 cells were used as controls. D-E: Western blot showing the expression of STAU1 and FABP4 protein in OA-treated, DMSO-treated, and untreated 3T3-L1 cells

STAU1 can bind to the CDS of mRNA and facilitate translation [10]. Considering that STAU1 is capable of binding to *FABP4* mRNA, we hypothesized that STAU1 may promote the ribosome occupancy of *FABP4*. To further elucidate the effects of STAU1 on *FABP4* RNA, we used a sucrose gradient to fractionate cytoplasmic mRNAs. Two siRNAs were used to downregulate STAU1 to avoid off-target effects in differentiated 3T3-L1 cells on day 4. Our results indicate that after the downregulation of STAU1, ribosome distribution changes significantly; polysome distribution is notably affected, while the percentage of monoribosomes increases significantly (Figure 3(a)). These results indicate that STAU1 may regulate ribosome and polysome stalling. We then compared the expression profile of *FABP4* mRNAs along each gradient. Expectedly, qRT-PCR revealed enrichment in endogenous *FAPB4* transcript levels in polysomal fractions from *STAU1*-knockdown cells compared with the levels of the controls. We also observed that *FABP4* mRNAs shifts towards the monoribosome fraction after STAU1 knockdown via both siRNAs (Figure 3(b)). STAU1 facilitates translation by unwinding the secondary structure of mRNA via p-UPF1, which is an ATPase-dependent helicase. Therefore, we used OA to retain UPF1 in a hyperphosphorylated state [20]. After 3T3-L1 cells were treated with OA, the expression of FABP4 protein increases significantly whereas *FABP4* mRNA levels do not change compared with the levels in the DMSO-treated controls (Figure 3(c-e)). These results indicate that STAU1 protein regulate the expression of FABP4 at the translational level.

### STAU1 attenuates adipogenesis by mediating the expression of FABP4

**Figure 4.**
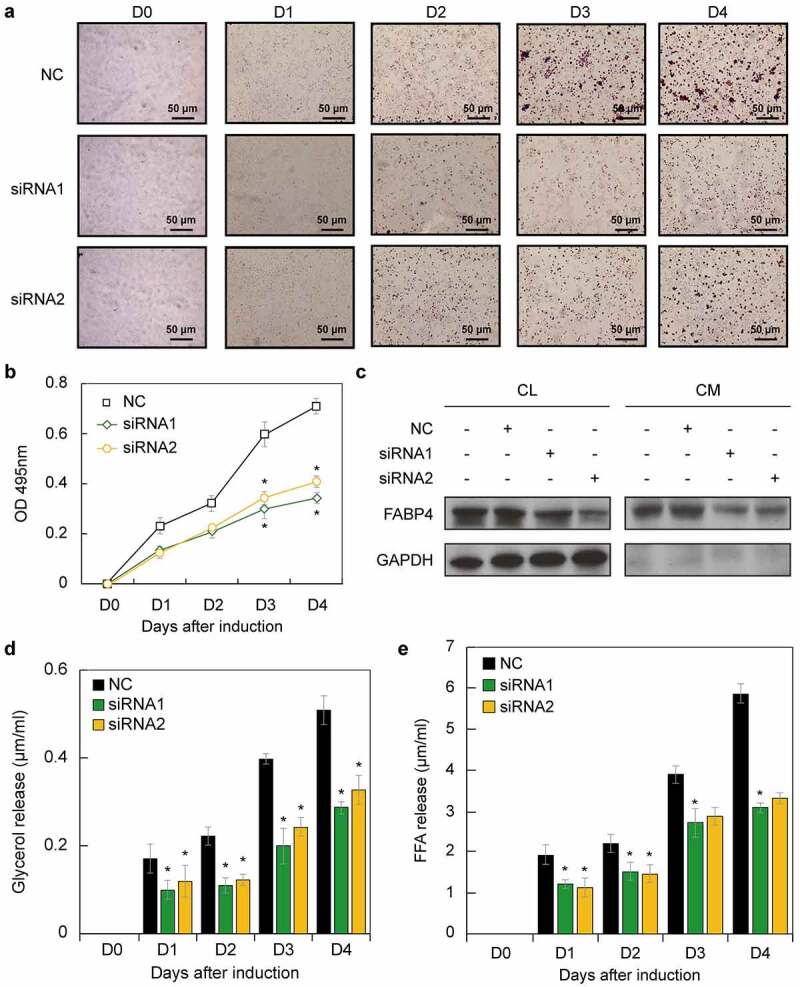
STAU1 regulates adipogenesis. A. Representative images of oil red O-stained 3T3-L1 cells at days 0, 1, 2, and 3. Scale bar, 50 µm. B. Lipid accumulation was assessed at 495 nm in 3T3-L1 cells stained with oil red O and de-stained with isopropyl alcohol (n =  3). C. Glycerol content in the cell culture medium (n =  3). F. FFA content in the cell culture medium (n =  3). FFA: free fatty acid. Values are expressed as means ± SEM *vs*. control group, * *P* < 0.05

FABP4 is a marker of adipogenesis. We sought to evaluate the function of STAU1 in lipid drop formation. Thus, we examined adipogenesis in STAU1-downregulated cells. Oil Red O was used to stain 3T3-L1 cells in which STAU1 was downregulated via siRNAs, and which were induced to differentiate into adipocytes on different days. Our results showed that lipid droplets in 3T3-L1 cells are significantly decreased after downregulation of STAU1 (Figure 4(a)). The absorption of 3T3-L1 cell lysates, obtained after oil red O staining, was then measured at 495 nm using a spectrophotometer. Our results revealed that adipogenesis is significantly decreased after downregulation of STAU1 (Figure 4(b)). Because FABP4 facilitates fatty acid transportation, we next assessed the secretion of FABP4 to evaluate fatty acid metabolism. 3T3-L1 cells were induced using the induction cocktail for 4 days and then transfected with STAU1 siRNAs. We found that FABP4 was present in both the cell lysates (CL) and cell medium (CM), while GAPDH, which is not a secretory protein, was not present in the CM. The levels of FABP4 levels in the CM and CL were significantly decreased after downregulation of STAU1, indicating that STAU1 may regulate fatty acid transport during adipogenesis. In addition, the glycerol and free fatty acid (FFA) content is markedly decreased in the CM after downregulation of STAU1 (Figure 4(d-e)). These results indicate that STAU1 regulates the lipid drop formation and lipid metabolism *in vitro*.

## *Overexpression of* STAU1 *in subcutaneous fat of mice increases weight and lipid metabolism*

**Figure 5.**
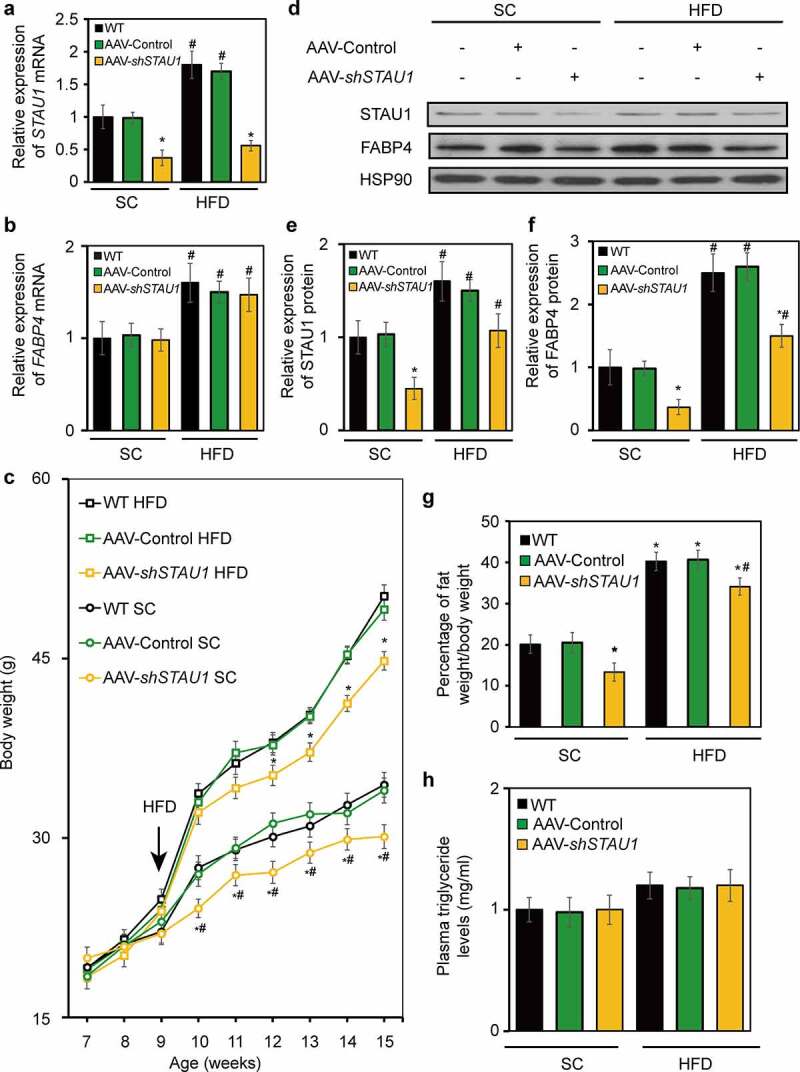
Effect of STAU1 knockdown on body weight and lipid metabolism in diet-induced obese mice. Gene expression analysis was performed using adipose tissue from mice fed standard chow (SC) and high-fat diet (HFD). The real-time PCR analysis of *STAU1* (a) and *FABP4* (b) mRNA expression relative to that of wild type SC-fed mice (3–5 animals per group). C: Bodyweight per mouse of groups fed HFD or SC diet and treated with AAV-control or AAV-*shSTAU1*. D: Western blot showing STAU1 and FABP4 expression in wild type SC- and HFD-fed mice after downregulation of STAU1. E-F: Quantification of STAU1 and FABP4 (f) protein levels relative to that of wild-type SC-fed mice. G: Nuclear magnetic resonance (NMR) analysis of body fat composition presented as percentage of body weight. *Represents a significant difference between wild-type group and AAV-control group or AAV*shSTAU1* group of SC-fed mice. #Represents a significant difference between wild-type group and AAV-control group or AAV*shSTAU1* group of HFD-fed mice

To examine the function of STAU1 *in vivo*, we generated an adeno-associated virus (AAV) encoding scrambled siRNA (AAV-Control) and siRNA designed according to the sequence of siRNA1 (AAV-*shSTAU1*), which targets *STAU1* mRNA. qRT-PCR show that AAV-*shSTAU1* knocks down the expression of *STAU1* mRNA (Figure 5(a)) in the adipose tissue of the HFD-fed mice by 62% compared with the expression levels of the wild-type (WT) mice fed a standard chow (SC) (5 ± 2 animals per group). Wild-type diet-induced obese mice that had been fed an HFD showed a 65% decrease in *STAU1* expression in the adipose tissue compared with that of WT mice. Considering that STAU1 regulates the expression of *FABP4 in vitro*, we performed qRT-PCR to measure *FABP4* levels in SC- and HFD-fed mice treated with AAV-control and AAV-*shSTAU1*. The expression of *FABP4* mRNA did not change in SC- and HFD-fed mice after downregulation of *STAU1* (Figure 5(b)). We then used western blotting to examine the expression of *STAU1* and *FABP4* in the adipose tissue of mice treated with AAV-control or AAV-*shSTAU1* compared with that of the control mice. In agreement with the *in vitro* results, the levels of STAU1 and FABP4 proteins were decreased in SC- and HFD-fed mice after expression of *STAU1* was knocked down by AAV-shSTAU1. Furthermore, the mRNA and protein levels of *STAU1* and *FABP4* were increased in HFD-fed mice compared with those in SC-fed mice. To characterize the functional impact of STAU1 knockdown, we analysed the bodyweight of SC- and HFD-fed mice. At week 10, the body weights of AAV-*shSTAU1* SC-fed mice were reduced compared with the body weights of SC-fed WT mice; this difference in the body weights of the mice groups was maintained throughout the remainder of our 8-week study. Half of the mice were switched to HFD at 9 weeks of age. After 6 weeks of consuming HFD, AAV-*shSTAU1* mice showed a significant increase in body weight compared with that of HFD-fed WT mice; however, downregulation of STAU1 decreased the body weights of AAV-*shSTAU1* mice fed HFD.

We then used quantitative nuclear magnetic resonance to examine whole-body fat mass in the different mouse cohorts. In both SC- and HFD-fed mouse cohorts, body fat content is significantly decreased in mice treated with AAV-*shSTAU1* compared with that of WT (Figure 5(g)). However, WT mice fed HFD demonstrate an almost four-fold increase in fat mass compared with that of SC-fed mice. Taken together, our data indicate that knockdown of *STAU1* robustly decreases the body weight and fat mass of HFD-fed mice. To investigate the potential physiological effects of *STAU1* knockdown, we analysed key plasma metabolic parameters in mice fed *ad libitum* or fasted overnight. As shown in Figure 5(h), triglyceride (TG) levels do not differ in SC-fed mice treated with AAV-*shSTAU1* or AAV-control. Furthermore, TG levels also remain unchanged in the different groups of HFD-fed mice. However, plasma TG levels of HFD-fed mice are increased compared with those of SC-fed mice. These results indicate that STAU1 regulates body weight and fat tissue accumulation in mice without affecting the levels of plasma TG.

## Discussion

Similar to other RNA-binding proteins, STAU1 participates in RNA surveillance, localization, and translation, as well as in alternative splicing during post-transcriptional processing [7,21,22]. During the translation of mRNA, secondary structures such as hairpins, stems, and loop can interfere with translation mechanisms [23]. In this study, we analysed the secondary structure of *FABP4* and found that STAU1 bound to the SBS of *FABP4* and facilitated *FABP4* translation. Knockdown of STAU1 affected the expression of FABP4 both *in vitro* and *in vivo*. We next examined the function of STAU1 in adipocytes and adipose tissue and found that STAU1 regulated adipogenesis and adipose development by regulating the expression of FABP4.

The expression of FABP4 is highly induced during adipogenesis and is transcriptionally controlled by PPARγ and C/EBPα [17]. The transcribed *FABP4* mRNA is spliced and transported to the cytoplasm to be translated into protein [24]. During these processes, secondary structures of mRNAs can greatly influence translation efficiency [25]. Certain structures within mRNAs can be recognized by RNA-binding proteins and they can affect the initiation and termination of translation or RNA decay [26]. In this study, we found four potential SBS in *FABP4* mRNA. Using PAR-CLIP, we verified that the SBS in position 3 could be recognized and bound by STAU1. This SBS in position 3 is located 110–220 nt from *FABP4* mRNA, which is located in the CDS region. It is possible that STAU1 binds to this region and recruits other RNA-binding proteins, such as regulator of nonsense transcripts 1 (UPF1). UPF1 is an ATP-dependent helicase that unwinds the double-stranded structure to facilitate the formation of polysome. Using sucrose density centrifugation, we found that knockdown of STAU1 inhibited polysome formation in *FABP4* mRNA. Distribution of *FABP4* mRNA was also altered by the knockdown of *STAU1*. We treated 3T3-L1 cells with OA to retain UPF1 in a hyperphosphorylated state. Our results indicate that maintaining UPF1 in a hyperphosphorylated state increases FABP4 expression at the protein level but not at the mRNA level. While expression of FABP4 was decreased by the downregulation of STAU1, the expression levels of PPARγ were increased. These results agree with those obtained by Tali et al., showing that FABP4 attenuated the expression of PPARγ and promoted its degradation [17].

FABP4 has a higher affinity and selectivity for long-chain fatty acids and facilitates the transportation of fatty acids to several cellular organelles [27]. Our analysis of the FFA content in the cell media indicated that the downregulation of STAU1 dramatically decreased the transportation of fatty acids; this result agreed with those obtained via oil red O staining. Downregulation of STAU1 also decreased the formation of lipid droplets within cells. These data show that STAU1 regulated adipogenesis *in vitro*. To downregulate the expression of STAU1 *in vivo* using AAV-encoded shRNA, we injected AAV into the subcutaneous fat tissues of mice. Our results indicate that downregulation of STAU1 in the fat tissues decreased the body weights and adipose tissue ratio in our mouse cohorts. However, the AAV-induced downregulation of STAU1 did not change FFA levels in mouse plasma. This may have occurred because the effects of a local injection are often not robust enough to induce systemic effects in mice.

STAU1 is a ubiquitously expressed dsRNA binding protein that can detect the secondary structure of mRNA [22,28]. The most important function of STAU1 occurs in Staufen-mediated mRNA decay (SMD). In the SMD pathway, STAU1 detects the double-stranded structure in the 3′ UTR of mRNA and recruits other RNA-binding proteins (RBPs) to degrade the mRNA [29]. STAU1 can also recognize the double-stranded structure in CDS and facilitate translation [30]. One study has shown that STAU1 can regulate the expression of MyoD at the translational level in quiescent MuSCs [7]. In this study, we have shown that STAU1 can regulate the translation of FABP4 during adipogenesis. Nonsense-mediated mRNA decay and SMD are competitive pathways [11]. STAU1 competes with UPF2 for binding to UPF1 and promotes the SMD pathway. Our results indicate that STAU1 may regulate alternative splicing (AS) events during adipogenesis (data not shown). In conclusion, we have shown that STAU1 regulates adipogenesis via binding to key mRNAs during adipogenesis and modifying these mRNAs by regulating AS, translation, or SMD.

RBPs play various roles in adipogenesis and obesity [31,32]. Among these RBPs, double-stranded RBPs are challenging to study because the binding of dsRNA-binding proteins is dependent on the secondary structure of RNA [33]. STAU1 is a typical dsRNA-binding protein; the RNAs bound by STAU1 differ in their fates and destinations. As described previously in this study, the many functions of STAU1 are mainly influenced by the position of SBS. Future studies may clarify which transcripts are bound by STAU1 during adipogenesis.

## Methods

### Chemicals, siRNAs, cell culture, and transfection

The mouse 3T3-L1 cells used in this study were acquired from the American Type Culture Collection (ATCC, Manassas, VA, USA). The 3T3-L1 cells were cultured to confluence in Dulbecco’s Modified Eagle’s Medium (DMEM; Gibco) supplemented with 10% foetal calf serum (FBS; Gibco, Grand Island, NY, USA). Okadaic acid (OA; Sigma–Aldrich) was dissolved in dimethyl sulphoxide (DMSO; Sangon) and used to treat the cells at a final concentration of 0.1 μM 4 days post-transduction. 3T3-L1 cells were then transiently transfected with siRNAs using Lipofectamine 3000 (Invitrogen) according to manufacturer’s instructions, at indicated time points, and harvested 2 days post-transfection. Total RNA was purified using TRIzol (Invitrogen), and total protein was extracted with cell lysis buffer (Thermo Fisher Scientific) containing a cOmplete™ ULTRA Tablets, Mini, *EASYpack* proteinase inhibitor (Roche).

Two siRNAs targeting STAU1 were synthesized by Sangon Biotech according to a previous study, siRNA1: 5′-r (CAACUGUACUACCUUUCCA) d (TT)-3′; siRNA2: 5′-r (AACGGUAACUGCCAUGAUA) d (TT)-3′ [18].

### Animals

All animal care and experimental procedures in this study were approved by the Animal Experimentation Ethics Committee of Xinjiang Medical University (approval number: 20,170,214–156). Animal study protocols complied with the ARRIVE guidelines and were carried out in accordance with the National Institutes of Health guide for the care and use of laboratory animals (NIH Publications No. 8023, revised 1978). Male C57Bl/6 J mice (4–5 weeks old) used in this study were obtained from the Experimental Animal Center of Xinjiang Medical University. Mice were housed in groups of 3–4 under standard housing conditions on a 12 h light/dark cycle and provided with *ad libitum* access to water and a standard chow diet until 9 weeks of age. Half of the mice were then switched to a high-fat diet (HFD), which was administered for an additional 11 weeks. Bodyweight and food intake were monitored weekly. Body fat composition was analysed using a Fat/Lean MiniQMR (Niumag, Suzhou, Jiangsu, China) at week 11 of the HFD.

## Adipogenesis, oil red O staining, and quantification of stained oil droplets

Murine 3T3-L1 cells were cultured in DMEM containing 10% FBS; the cell suspension was adjusted to approximately 1 × 10^5^ cells/mL. After 48 h, the cells were treated with an induction cocktail containing 10 μg/mL insulin (Sigma–Aldrich), 115 μg/mL 3-isobutyl-1-methylxanthine (Sigma–Aldrich), and 3.9 μg/mL dexamethasone (Thermo Fisher Scientific). The cells were then allowed to incubate until differentiation was observed, and the degree of differentiation was assessed at different time points.

To detect the accumulated lipid droplets, differentiated 3T3-L1 cells were washed three times with PBS and fixed using 4% paraformaldehyde (Thermo Fisher Scientific) for 2 h at 22–27°C. The cells were then stained with 60% oil red O (Thermo Fisher Scientific) for 30 min and washed with PBS. To quantitate the stained triglycerides, the cells were dissolved in isopropanol for 10 min and then evaluated at 495 nm with an Agilent Cary 3500 spectrophotometer (Agilent Technologies, Santa Clara, CA, USA).

### Quantitative real-time PCR

Total RNA was harvested from cells or tissues at different time points. Quality and the concentration of RNA were measured using a Nanodrop Spectrophotometer (Thermo Fisher Scientific, Waltham, MA, USA). cDNA was prepared using a SuperScript III Reverse Transcription Kit (Thermo Fisher Scientific) according to the manufacturer’s instructions. qRT-PCR was performed on an ABI 7500 (Applied Biosystems, Waltham, MA, USA) using SYBR-Green PCR kit I (Applied Biosystems) according to the manufacturer’s instructions. The primers used for *STAU1, FABP4, PPARγ, CEBP/α, HSP90*, and *GAPDH* mRNA have been previously described [34].

### Western blotting

A total of 50 μg of each of the samples was loaded into each lane of 12% SDS-PAGE gels, transferred to nitrocellulose membranes (Bio-Rad, Hercules, CA, USA), blocked with 5% bovine serum albumin, and probed with primary antibodies overnight. Antibodies were used at concentrations recommended by their respective manufacturers. For this procedure, we used primary antibodies against HSP90 (Cat: BM4191, Boster, Wuhan, Hubei, China), mouse STAU1 (Cat: ab73478, Abcam, Cambridge, MA, USA), mouse FABP4 (Abcam, Cambridge, MA, USA), mouse PPARγ (Cat: ab66682, Abcam, Cambridge, MA, USA), and mouse CEBP/α (Cat: sc-365,318, Santa Cruz, Santa Cruz, CA, USA). The membranes were first incubated with the primary antibodies overnight at 22–27°C and then with horseradish peroxidase-conjugated secondary antibodies (Cat: BA1077, Goat Anti-Mouse IgG (H + L) Secondary Antibody, HRP Conjugate; Cat: BA1054, Goat Anti-Rabbit IgG (H + L) Secondary Antibody, HRP Conjugate, Boster, Wuhan, Hubei, China). The signal was detected via chemiluminescence using an ECL kit (Bio-Rad). Densitometric analysis was performed using Image Lab software (Bio-Rad, Hercules, CA, USA).

### Sucrose density centrifugation

3T3-L1 cells were lysed using RIPA buffer. The extracts were cleared through centrifugation for 10 min at 12,000 × *g* and loaded onto a continuous 15%–40% sucrose gradient. Fifteen fractions were collected after centrifugation for 16 h at 83,000 *× g*. To assess polysome proliferation, the absorption of each fraction was determined at 254 nm by an Agilent Cary 3500 spectrometer. To determine the distribution of *FABP4* mRNA, total RNA was extracted from each fraction using a phenol: chloroform: isopropanol mixture.

### *RNA immuno-precipitation and photoactivatable ribonucleotide enhanced crosslinking* and *immunoprecipitation*

RNA immune-precipitation (RIP) was conducted using an RNA Immunoprecipitation Kit (Millipore, Billerica) per manufacturer’s instructions. Lysed 3T3-L1 cells were incubated overnight with 10 μL of STAU1 antibody and then precipitated using 30 μL A/G agarose beads (GE, Little Chalfont, Buckinghamshire, UK). After precipitation, RNA was extracted using the phenol: chloroform: isoprene mixture.

3T3-L1 cells at 80% confluence were then evaluated using a PAR-CLIP assay. For this, the cells were treated with 100 μM 4-thiouridine (SU, Sigma–Aldrich), incubated at 37°C for 16 h, and then irradiated with 150 mJ/cm^2^, 365 nm ultraviolet light in a UV crosslinker (CL-1000 L, UVP, USA) to facilitate crosslinking of RNA with STAU1. Cellular lysates were prepared and incubated with RNase T1 at the final concentration of 1 U/μL in a water bath for 15 min at 22°C. Protein G magnetic beads were incubated with anti-STAU1 antibody (Cat: ab73478, Abcam, Cambridge, MA, USA) or IgG. Next, the mRNA-STAU1 complex was isolated from the lysate by immunoprecipitation. A second RNase T1 digestion ensured that only the RNA segment that was bound and protected by STAU1 was detected. RNA was extracted using a phenol: chloroform: isoprene mixture. Primers for each section of the *FABP4* mRNA were designed, and exact binding sites of STAU1 protein and *FABP4* mRNA were detected via qPCR.

### Administration of recombinant AAV vectors

Single-stranded AAV vectors were produced via triple transfection of human embryonic kidney 293 cells and purified using a CsCl-based gradient described previously [35]. The AAV-*shSTAU1* was targeted to mouse *STAU1* mRNA carrying the same sequence as siRNA1 used *in vitro* as described previously in the ‘Chemicals, siRNAs, cell culture, and transfection’ section of the Methods.

Mice were anesthetized with ketamine (100 mg/kg) and xylazine (10 mg/kg). A laparotomy was performed for the intraepididymal (intrae) delivery of white adipose tissue (WAT). To distribute the vector in the whole depot, each epididymal fat pad was injected twice with 500 μL of AAV-containing solution.

### Plasma analysis

Plasma TG and free fatty acid levels were measured using a commercial kit (Cat: KT37563, MSK) and half micro-test (Cat: KT21006, MSK), respectively, according to the manufacturer’s instructions.

### Statistical analysis

The Student’s *t*-test (two-tailed) and one-way ANOVA were conducted to analyse the *in vivo* and *in vitro* data using SPSS 19.0 (IBM, New York, USA). Data are expressed as the mean ±standard deviation. The hypotheses were tested by calculating the 95% confidence interval or by one-way or two-way ANOVA statistical tests. *P* < 0.05 was considered statistically significant.
